# Editorial: Blood microbiota in health and disease

**DOI:** 10.3389/fcimb.2023.1187247

**Published:** 2023-03-22

**Authors:** Mehmet Demirci, A. Suat Saribas, Seyed Davar Siadat, Bekir Sami Kocazeybek

**Affiliations:** ^1^ Department of Medical Microbiology, Faculty of Medicine, Kirklareli University, Kirklareli, Türkiye; ^2^ Department of Medical Microbiology, Cerrahpasa Faculty of Medicine, Istanbul University-Cerrahpasa, Istanbul, Türkiye; ^3^ Microbiology Research Center (MRC), Pasteur Institute of Iran, Tehran, Iran; ^4^ Mycobacteriology and Pulmonary Research Department, Pasteur Institute of Iran, Tehran, Iran

**Keywords:** blood microbiota, blood microbiome, health, disease, pathogenesis, immune response, pathophysiology, mechanism

In this Research Topic of Frontiers in Cellular and Infection Microbiology journal, We have tried to emphasise studies on blood microbiota and its different effects on health and disease. Blood acts as a liquid medium carrying the elements necessary for host life. In recent years, the concept of human blood microbiota has been developed and this has caused serious debates as it may shelve the idea that “blood is a sterile environment” that has been in existence for years. Although there are different hypotheses that the blood microbiota originates from the gut microbiota, its origin is not clearly understood because it contains different phyla (Castillo et al., 2019). In today’s medicine, where faecal microbiota transplantation is being considered for treatment, blood transplantation is much more easily feasible. If the effects of the blood microbiota on health and disease can be demonstrated, intervention here may be possible (Almeida et al., 2022). Therefore, the aim of this Research Topic was to provide an up-to-date summary on recent findings on the role of the blood microbiota and to explore relationships between the blood microbiota and diseases. This Research Topic also acted as a key initiative towards facilitating a discussion on the role of blood microbiota and its interactions with the immune system during health and disease.

In the study of Khan et al., investigated the blood microbial composition of patients with acute coronary syndrome and chronic coronary syndrome using V3-V4 region 16S rRNA sequencing. They reported after linear discriminant analysis, *Proteobacteria* and *Acidobacteriota* phyla in the acute coronary syndrome group and *Firmicutes* phylum and *Lactobacillus* genus in the chronic coronary syndrome group were significantly different from healthy controls. They also reported that atherosclerosis patients and healthy controls have distinct blood microbiota diversity. Further investigations are still needed to determine the effect on the pathogenesis of atherosclerosis and to confirm the results.

Hidi et al., revealed the microbiota found in healthy vessel walls. In their study, they analysed femoral artery samples taken from donors in multiple organ donations using V3-V4 region 16S rRNA sequencing. They reported that the most abundant phyla in these samples were *Proteobacteria*, *Firmicutes* and *Actinobacteria*, and at the genus level, *Staphylococcus*, *Corynebacterium*, *Pseudomonas*, *Bacillus*, *Acinetobacter* and *Propionibacterium*. Bacteria such as *Roseburia* and *Ruminococcus*, which are more common in the gut microbiota of healthy controls compared to atherosclerosis patients, were detected in most of the samples. They stated that the composition of the human arterial wall microbiota has a unique microbiota with significant differences compared to other parts of the human body. They reported that these data will form the basis for vascular allograft transplantation and may contribute to the success of this transplantation.

Szabó et al., reported that although the existence of a “eubiotic” blood microbiota in healthy humans has been discussed, the existence of a dysbiosis state in the blood of patients with sepsis, especially in nosocomial infections, has not been discussed. Therefore, the authors compared the composition of the blood microbiota of adult septic patients with community-acquired infections with non-septic controls using V3-V4 region 16S rRNA sequencing. As a result of blood microbiota analysis, they reported that at the phylum level, *Firmicutes* were detected in septic patients with significantly lower abundance and *Proteobacteria* with significantly higher abundance. At the genus level, they reported that the abundance of *Pseudomonas*, *Micrococcus* and *Enhydrobacter* was significantly higher in the blood microbiota of septic patients, but they could not detect these bacteria as etiopathogens by classical methods such as blood culture. They reported that this change in blood microbiota in sepsis patients occurred as a result of the inflammatory process occurring during sepsis, leading to damage of different barrier layers of the body.

Finally, Tsafarova et al., reported that although blood microbiota has been proven in healthy individuals in recent years, it is still an enigma. They stated that although there are live microbial forms or a rich nucleic acid biodiversity in the blood, the morphology and proliferation cycle of this microbiota are not clear. For this reason, they focused on studying the life cycle of blood microbiota by next generation sequencing, light and electron microscopy (TEM and SEM) and culture. Similarly to Panaiotov et al. (2018), Tsafarova et al. reported that the results of electron microscopy and culture confirmed that the blood microbiome represents living structures rather than debris resulting from the degradation of blood elements such as lipids or haemoglobin complexes. In freshly collected and cultured blood, they found that the blood microbiota undergo complex life cycles involving different morphological transformations. they reported that they observed a rare “cell within a cell”-like proliferation phenomenon. they were interested in the intracellular mechanisms of blood microbiota proliferation in healthy individuals and presented basic data on this subject.

In conclusion, the available evidence both proves the existence of blood microbiota and supports its effects in health and disease. We hope that the Research Topic presented in this issue will change the debate on the blood microbiota and help to accelerate large-scale studies focusing on its effects.

## Author contributions

MD - writing the manuscript; AS, SS, and BK - manuscript editing and approval. All authors listed have made a substantial, direct, and intellectual contribution to the work and approved it for publication.

## References

[B1] Almeida C. Oliveira R. Baylina P. Fernandes R. Teixeira F. G. Barata P. (2022). Current trends and challenges of fecal microbiota transplantation-an easy method that works for all? Biomedicines 10 (11), 2742. doi: 10.3390/biomedicines10112742 36359265 PMC9687574

[B2] Castillo D. J. Rifkin R. F. Cowan D. A. Potgieter M. (2019). The healthy human blood microbiome: Fact or fiction? Front. Cell Infect. Microbiol. 9. doi: 10.3389/fcimb.2019.00148 PMC6519389 31139578

[B3] Panaiotov S. Filevski G. Equestre M. Nikolova E. Kalfin R. (2018). Cultural isolation and characteristics of the blood microbiome of healthy individuals. Adv. Microbiol. 8, 406–421. doi: 10.4236/aim.2018.85027

